# Drains and Diphtheria

**Published:** 1898-12-24

**Authors:** 


### The Hospital

A Journal of

The Medical Sciences and Hospital Administration.

Vol. XXV.—No. 639.] Edited by Sir Henry Burdett, K.C.B., and Solomon C. Smith, M.D., M.R.C.P. [Dec. 24, 1898.

### Drains and Diphtheria.

A case was tried recently before Mr. Justice Lawrance in which a tailor sued his landlord for damages for breach of agreement for neglecting to provide a sanitary water-closet to a house which he rented from the defendant. The case to a large extent hinged upon the claim that the diphtheria from which the family of the plaintiff had suffered, and from which two of his children had died, had been caused by the defective condition of the drains. On this question there was a direct conflict of medical evidence. Dr. Dudfield is reported to have "expressed his opinion, from what he had heard in evidence, that the little girls had caught diphtheria from the bad drains," while Dr. Bately, medical officer of health, and Sir Hugh Beevor, M.D., both expressed their opinion that the diphtheria which attacked the girl first affected, and which about the same time attacked a school-fellow of hers, who also died, was the result of school infection, and was not caused by the drains, as suggested. In the end the jury gave a verdict for the plaintiff, and assessed the damages at £250.

The question of the source of diphtheria is a very important and, so far as individual cases are concerned, a most difficult one. In fact it must always be to a large extent a matter of opinion in what manner an individual catches an infectious disease, especially a disease which is widely spread; and seeing the varying parts played by the two great influences involved in the etiology of diphtheria, namely, infection and predisposition, there must always be considerable room for conflict of opinion in such a case as the one we have referred to. But if we put the individual on one side and consider the incidence of the disease on large groups of people, we can hardly doubt that bad drainage has an important influence in encouraging the prevalence, although not, perhaps, in directly causing the infection of diphtheria.

All the old clinical experience pointed to bad sewers and accumulations of offensive refuse, and especially to a combination of insanitation with dampness of house, as being among the most important conditions predisposing to the prevalence of diphtheria. Lately, however, the great development of diphtheria which has occurred in the course of a period during which the country has undoubtedly been undergoing great sanitary improvements, and while other diseases which are certainly encouraged by insanitary conditions have been rapidly diminishing, has thrown a doubt upon the direct relationship of diphtheria with such insanitary surroundings. Nevertheless, the old observations remain true, and new ones are being constantly added, which tend to show that exposure to foul emanations is a predisposing cause of the disease. The explanation is very probably the one given by Sir Richard Thorne, who says such emanations are productive of sore throats, and that a morbid condition of the fauces affords a soil favourable to the lodgement and maintenance of the infection of diphtheria, but of the fact that in this or some other way there is a relation between diphtheria and drain effluvia we cannot doubt. An interesting observation bearing upon this occurs in the last annual report of Mr. Warry, medical officer of health for Hackney. During the year 1897 he had the drainage of every house in which certain infectious diseases had occurred carefully examined, and the result was that the percentage of houses with defective drains was found to be as follows in the different diseases: Enteric fever, 29·8; diphtheria, 27·8; scarlatina, 18·7; erysipelas, 18·1. Thus it appears that in frequency of connection with defective drains diphtheria stood along with enteric fever, and was far ahead both of scarlet fever and erysipelas, so that we must either imagine that the two latter diseases fell with undue frequency upon the inhabitants of healthy houses, which is extremely unlikely, or that diphtheria occurred in large excess in houses whose drains were bad. It must be remembered also that the argument which has been so strongly urged of late to the effect that the spread of diphtheria alongside of and almost parallel with a marked improvement in the sanitary condition of the country, is no proof that diphtheria has nothing to do with insanitation. What the facts really seem to prove is that the extension of diphtheria coincidently with the improvement of the drains is due to the intrusion of some entirely new influence, namely, that of the schools, which was not in such wide operation before. Hence, in the case we have spoken of, possibly both sets of medical witnesses were right, each in their own way. It is true that children are often exposed to the infection of diphtheria in schools, but it is probably also true that whether the individual exposed will develop the diseases or not depends much upon the condition of his health and of his throat—and that is where the drains come in.

